# New Books

**Published:** 2011-03

**Adapting to the Impacts of Climate Change**

America’s Climate Choices: Panel on Adapting to the Impacts of Climate Change; National Research Council

Washington, DC:National Academies Press, 2010. 292 pp. ISBN: 978-0-309-14591-6, $49.95

**Advancing the Science of Climate Change**

America’s Climate Choices: Panel on Advancing the Science of Climate Change; National Research Council

Washington, DC:National Academies Press, 2010. 528 pp. ISBN: 978-0-309-14588-6, $49.95

**America’s Food: What You Don’t Know About What You Eat**

Harvey Blatt

Cambridge, MA:MIT Press, 2011. 384 pp. ISBN: 978-0-262-51595-5, $18.95

**Beyond Resource Wars: Scarcity, Environmental Degradation, and International Cooperation**

Shlomi Dinar, ed.

Cambridge, MA:MIT Press, 2011. 320 pp. ISBN: 978-0-262-01497-7, $50

**Coming Clean: Information Disclosure and Environmental Performance**

Michael E. Kraft, Mark Stephan, Troy D. Abel

Cambridge, MA:MIT Press, 2011. 264 pp. ISBN: 978-0-262-01495-3, $50

**Conservation Refugees: The Hundred-Year Conflict between Global Conservation and Native Peoples**

Mark Dowie

Cambridge, MA:MIT Press, 2011. 336 pp. ISBN: 978-0-262-51600-6, $15.95

**Encyclopedia of Environmental Health**

Jerome Nriagu

St. Louis, MO: Elsevier, 2011. 5,000 pp. ISBN: 978-0-444-52273-3, $1,800

**Environmental Biotechnology: Theory and Application**

Gareth G. Evans, Judy Furlong

Weinheim:Wiley-VCH Verlag GmbH & Co., 2010. 290 pp. ISBN: 978-0-470-68417-7, $79.95

**Environmental Inequalities Beyond Borders: Local Perspectives on Global Injustices**

JoAnn Carmin, Julian Agyeman, eds.

Cambridge, MA:MIT Press, 2011. 256 pp. ISBN: 978-0-262-01551-6, $50

**Global Catastrophic Risks**

Nick Bostrom, Milan M. Cirkovic, eds.

New York:Oxford University Press, 2011. 576 pp. ISBN: 978-0-19-960650-4, $25.95

**Informing an Effective Response to Climate Change**

America’s Climate Choices: Panel on Informing Effective Decisions and Actions Related to Climate Change

Washington, DC:National Academies Press, 2010. 348 pp. ISBN: 978-0-309-14594-7, $49.95

**Lead and Public Health, Vol 10**

Paul Mushak

St. Louis, MO:Elsevier, 2011. 650 pp. ISBN: 978-0-444-51554-4, $210

**Limiting the Magnitude of Future Climate Change**

America’s Climate Choices: Panel on Limiting the Magnitude of Future Climate Change; National Research Council

Washington, DC:National Academies Press, 2010. 276 pp. ISBN: 978-0-309-14597-8, $49.95

**Living in Denial: Climate Change, Emotions, and Everyday Life**

Kari Marie Norgaard

Cambridge, MA:MIT Press, 2011. 288 pp. ISBN: 978-0-262-01544-8, $50

**Paths to a Green World, 2nd ed.: The Political Economy of the Global Environment**

Jennifer Clapp, Peter Dauvergne

Cambridge, MA:MIT Press, 2011. 336 pp. ISBN: 978-0-262-51582-5, $27

**Review of Closure Plans for the Baseline Incineration Chemical Agent Disposal Facilities**

Committee to Review and Assess Closure Plans for the Tooele Chemical Agent Disposal Facility and the Chemical Agent Munitions Disposal System; National Research Council

Washington, DC:National Academies Press, 2010. 106 pp. ISBN: 978-0-309-15858-9, $28.75

**Sustainability or Collapse? An Integrated History and Future of People on Earth**

Robert Costanza, Lisa J. Graumlich, Will Steffen, eds.

Cambridge, MA:MIT Press, 2011. 517 pp. ISBN: 978-0-262-51597-9, $22

**Water Sustainability A Global Perspective**

J.A.A. Jones

New York:Oxford University Press, 2011. 256 pp. ISBN: 978-1-4441-0488-2, $59.99

